# Machine learning analysis of gene expression data reveals novel diagnostic and prognostic biomarkers and identifies therapeutic targets for soft tissue sarcomas

**DOI:** 10.1371/journal.pcbi.1006826

**Published:** 2019-02-20

**Authors:** David G. P. van IJzendoorn, Karoly Szuhai, Inge H. Briaire-de Bruijn, Marie Kostine, Marieke L. Kuijjer, Judith V. M. G. Bovée

**Affiliations:** 1 Department of Pathology, Leiden University Medical Center, Leiden, The Netherlands; 2 Department of Cell and Chemical Biology, Leiden University Medical Center, Leiden, The Netherlands; 3 Centre for Molecular Medicine Norway, University of Oslo, Oslo, Norway; Ottawa University, CANADA

## Abstract

Based on morphology it is often challenging to distinguish between the many different soft tissue sarcoma subtypes. Moreover, outcome of disease is highly variable even between patients with the same disease. Machine learning on transcriptome sequencing data could be a valuable new tool to understand differences between and within entities. Here we used machine learning analysis to identify novel diagnostic and prognostic markers and therapeutic targets for soft tissue sarcomas. Gene expression data was used from the Cancer Genome Atlas, the Genotype-Tissue Expression project and the French Sarcoma Group. We identified three groups of tumors that overlap in their molecular profiles as seen with unsupervised t-Distributed Stochastic Neighbor Embedding clustering and a deep neural network. The three groups corresponded to subtypes that are morphologically overlapping. Using a random forest algorithm, we identified novel diagnostic markers for soft tissue sarcoma that distinguished between synovial sarcoma and MPNST, and that we validated using qRT-PCR in an independent series. Next, we identified prognostic genes that are strong predictors of disease outcome when used in a k-nearest neighbor algorithm. The prognostic genes were further validated in expression data from the French Sarcoma Group. One of these, *HMMR*, was validated in an independent series of leiomyosarcomas using immunohistochemistry on tissue micro array as a prognostic gene for disease-free interval. Furthermore, reconstruction of regulatory networks combined with data from the Connectivity Map showed, amongst others, that HDAC inhibitors could be a potential effective therapy for multiple soft tissue sarcoma subtypes. A viability assay with two HDAC inhibitors confirmed that both leiomyosarcoma and synovial sarcoma are sensitive to HDAC inhibition. In this study we identified novel diagnostic markers, prognostic markers and therapeutic leads from multiple soft tissue sarcoma gene expression datasets. Thus, machine learning algorithms are powerful new tools to improve our understanding of rare tumor entities.

## Author summary

Soft-tissue sarcomas are a group of rare cancers that can be challenging to diagnose and treat. The morphology of the different soft-tissue sarcoma subtypes can overlap and the prognosis differs significantly between, and also within, the different subtypes. Moreover, targeted therapies are often not available. In this study we used transcriptome sequencing data from The Cancer Genome Atlas, containing 206 soft-tissue sarcoma samples which we analyzed using different machine learning algorithms to gain novel insights. When possible, we verified our findings in independent datasets or in cell lines. First, we found that both synovial sarcomas and malignant peripheral nerve sheath tumors show the largest overlap with normal tissue derived from the nervous system. This link with neural differentiation for synovial sarcoma was not well established until now. Second, genes were identified whose expression could be used to differentiate between the different soft-tissue sarcomas where the morphology overlaps. Third, novel prognostic genes were identified for separate subtypes. One gene, *HMMR*, which we found as a strong prognostic gene for leiomyosarcoma, was verified with immunohistochemistry on samples from our archives. Last, using a network analysis new potential therapies were identified. HDAC inhibitors were identified as a potential strong therapy for sarcomas, including leiomyosarcomas, which we verified in cell lines.

## Introduction

Soft tissue sarcomas are rare malignancies arising in the tissues that connect, support and surround other body structures, such as fat or muscle [1]. Soft tissue sarcomas annually affect approximately one per 50 million population, and represent <1% of all malignant tumors [2]. Soft tissue sarcomas can display different lines of differentiation, and as such are classified based on the tissue that they resemble most. More than 50 different subtypes have been described in the WHO classification. Even though these subtypes differ in prognosis and treatment, there is considerable morphological overlap between the different subtypes, making differential diagnosis both difficult and important. For instance, synovial sarcoma (SS) and malignant peripheral nerve sheath tumor (MPNST) can be morphologically identical, while also their immunohistochemical profile can overlap, making molecular testing for the presence of the SS specific SS18-SSX fusion essential for the final diagnosis (which is laborious and time consuming). Over the last years there have been many large genetic studies generating open accessible gene expression datasets of sarcomas. One of the biggest soft tissue sarcoma sequencing projects to date is the Cancer Genome Atlas (TCGA), which recently published a detailed analysis of the driving mutations in these cancers [3]. This data can be leveraged and analyzed with machine learning methodologies to better understand soft tissue sarcoma biology. Machine learning has been used previously to study gene expression patterns. Especially unsupervised algorithms, such as Principal Component Analysis (PCA) and more recently t-Distributed Stochastic Neighbor Embedding (t-SNE), have been successfully used in gene expression studies to classify cancer patients [4]. Moreover, for classification of tumors, supervised algorithms such as random forest have been used previously. Gene expression signatures were shown to be effective at classifying breast cancer [5]. Later, it was shown that microRNA expression patterns could be used to distinguish between a number of different tumor subtypes, ranging from brain to colorectal cancer [6]. More recently, random forest analyses were used on DNA-methylation data to classify different brain tumor subtypes. The advantage of the latter is that it can be performed on paraffin embedded material [7,8].

Previously the French Sarcoma Group used a machine learning approach on a large cohort of soft tissue sarcomas to verify a set of 67 genes (CINSARC), identified using differential expression analysis, that effectively predicted metastatic outcome in soft tissue sarcomas [9]. The identified CINSARC genes were more recently found to have prognostic value for other tumor types as well, such as breast cancer [10]. The CINSARC genes are mostly associated with cell proliferation and therefore lack tumor subtype specificity. Another approach to identify prognostic genes was used by the Pathology Atlas to identify tumor subtype specific prognostic genes. However, soft tissue sarcomas were not analyzed in this study [11].

In this study we used machine learning on open accessible expression data from soft tissue sarcomas to elucidate differences between and within the different entities. First, we investigated the overlap of gene expression patterns of soft tissue sarcomas with gene expression patterns of human tissues without malignancies from the GTEx project [12] using clustering with PCA and a deep neural network. Second, we identified novel diagnostic markers using a random forest approach. Third, we identified tumor subtype specific prognostic genes and showed, using a k-nearest neighbor analysis, that the identified prognostic genes are predictive of the metastasis-free interval. Last, we analyzed differential expression in the context of a regulatory network to identify novel therapies. We demonstrate that machine learning can be a powerful tool to identify novel diagnostic and prognostic biomarkers, as well as therapeutic targets, which will improve our understanding of rare soft tissue sarcomas.

## Materials and methods

### Ethics statement

All the specimens were coded and handled according to the ethical guidelines described in the Code for Proper Secondary Use of Human Tissue in the Netherlands of the Dutch Federation of Medical Scientific Societies as reviewed and approved by the LUMC ethical board (B17.036).

### Expression data

The Cancer Genome Atlas (TCGA) RNA-seq count data was downloaded (February 2018) from the NIH GDC data portal (portal.gdc.cancer.gov/). All clinical data corresponding to the soft tissue sarcoma samples in the TCGA was recently revised by the Cancer Genome Atlas Research Network which resulted in 206 revised cases with clinical data (from the original 261 cases in the TCGA) [3]. Soft tissue leiomyosarcoma (STLMS) was the most common sarcoma type with 53 samples and included cases of grade 1 (n = 11), grade 2 (n = 35) and grade 3 (n = 7) according to the Fédération Nationale des Centres de Lutte Contre le Cancer (FNCLCC) grading system. In addition, there were 27 uterine leiomyosarcoma (ULMS) cases. Furthermore, the TCGA included 50 dedifferentiated liposarcomas (DDLPS), 44 undifferentiated pleomorphic sarcomas (UPS), 17 myxofibrosarcomas (MFS), 10 synovial sarcomas (SS, both monophasic and biphasic) and 5 malignant peripheral nerve sheath tumors (MPNST).

Second, the Genotype-Tissue Expression (GTEx) data (v7) was downloaded (gtexportal.org) with corresponding annotations. The data consisted of transcriptome sequencing read counts for 9662 samples. The GTEx data included expression data for 31 different tissue types (S1 Table). Third, DDLPS (n = 62) and leiomyosarcoma (LMS) (n = 84) expression array data from the French Sarcoma Group was downloaded from GEO (ncbi.nlm.nih.gov/geo), deposited under accession number GSE21050 (public in June 2010), using GEOquery (v3.6) in R [13].

### Normalization of expression data

Genes with low expression (transcriptome sequencing read counts: cpm < 2; expression array: relative measured unit < 2) in all samples were removed. Thereafter, transcriptome sequencing read count and expression array data were normalized using Limma (v3.6) R package. For normalization, the weighted trimmed mean of M-values was used [14]. Last, the data was log2 transformed and analyzed further. When indicated, data was combined and normalized. Where indicated samples were randomly subdivided into groups using the “sample” function in R.

### Machine learning analysis

For the deep neural network TensorFlow (v1.6) was used in combination with the Keras (v2.1.4) R package to design a neural network with one converging invisible layer. t-SNE was performed using the Rtsne (v0.13) R package. For t-SNE analysis a perplexity of 60 and a theta of 0.5 were used. Random forest analysis was performed on the normalized TCGA expression data. Data were analyzed according to Breiman’s random forest algorithm, using the randomForest (v4.6) R package. Variable importance in the random forest analysis was calculated based on the Gini index, which is a measurement of variance for a given variable. For k-Nearest Neighbor analysis the Caret (v6.0) R package was used. To resample the data, the “repeatedcv” option was used and k = 1–30 were tested.

### Enrichment analysis

The EnrichR (v1.0) R package was used for Gene Ontology (GO) term enrichment analysis. GO terms were selected from the “GO biological processes 2015” database and had adjusted p values lower than 1e-4.

### Kaplan-Meier analysis

As readout disease-free interval (DFI) was used, which was previously described as a strong measurement of outcome in soft tissue sarcomas [15]. DFI is the time until relapse, including distant metastasis and loco-regional recurrence. Prognostic genes were identified using the maxstat (v0.7) R package. Maxstat determined the maximal rank statistic using a LogRank analysis, to determine the optimal gene expression cut-off. P values were calculated according to the Streitberg algorithm [16]. Version 18 of the Human Protein Atlas data was downloaded to cross-check prognostic genes identified in other tumor types (proteinatlas.org/about/download). This dataset included genes and their association with disease outcome in common cancer types.

### Immunohistochemistry and analysis

Immunohistochemistry (IHC) was performed on one existing tissue microarray (TMA) and one newly constructed TMA. The TMA was constructed as previously described by our group [17]. Clinicopathological details are summarized in S2 Table. In total, seventy leiomyosarcomas could be scored for HMMR protein expression and had available clinicopathological information. The cases originated from two cohorts: the first contained 32 cases that could be scored and has been previously described by our group [17], the second consisted of 38 cases that could be scored. IHC was performed simultaneously on all cases to enable comparison between the cohorts. The 70 cases consisted of 43 females and 27 males, with a mean age of 62 years at diagnosis. Five patients had uterine LMS, the rest were soft tissue LMS. Soft tissue LMS were graded according to the FNCLCC grading system, including 10 grade 1, 23 grade 2, 31 grade 3 and for 1 grading was not available. HMMR was detected with a polyclonal rabbit antibody (Sigma-Aldrich; HPA040025). The HMMR antibody was titrated on normal testis tissue, the optimal antibody dilution was found to be 1:1000 in PBS/1%BSA/5%/non-fat dry milk. Microwave antigen retrieval was performed using citrate (pH 6.0) and immunohistochemistry was performed according to standard protocols [18]. Scoring was performed using ImageJ (v1.5) in which color deconvolution was used to separate haematoxylin and 3,3'-Diaminobenzidine (DAB) staining. Haematoxylin was used to identify the core and intensity of the DAB was quantified and compared between cores. A cut-off score of 20 was used to define high and low expressing cores. The second cohort was also scored manually by a pathologist (JVMGB) blinded towards clinicopathological data and results of the automatic scoring, in which staining intensity was scored as weak (1), moderate (2) or strong (3). For the analysis, the average of the three cores per tumor were used.

### Quantitative reverse transcriptase Polymerase Chain Reaction (qRT-PCR)

Frozen tissue from five SS and four MPNSTs was retrieved from our archive and anonymized. All selected MPNSTs were either associated with a nerve, were NF1 related or had reported loss of H3K27me3 at immunohistochemistry [19,20]. All selected synovial sarcomas were proven to be positive for the SS18-SSX translocation. RNA was isolated using the Direct-zol RNA isolation kit (Zymo research). cDNA was made using iScript cDNA Synthesis Kit (Bio-Rad). Real-time PCR was performed using Sybr Green (Bio-Rad) on a CFX384 real-time PCR Detection System (Bio-Rad). Real-time PCR Ct values were normalized to housekeeping gene *HPRT1* expression. The following primers were used, noted as 5’ to 3’:

*NEURL1*_Fw GCATCCTCGGCTCCACTATC

*NEURL1*_Rv CTGAGCAAGGGGTCAGACAG

*SCD*_Fw CTTGCGATATGCTGTGGTGC

*SCD*_Rv CCGGGGGCTAATGTTCTTGT

*NPAS1*_Fw CAGCTGCTACCAGTTTGTCCAC

*NPAS1*_Rv ACCCTTGTCCAGCAAGTCCAC

*HPRT1*_Fw TGACACTGGCAAAACAATGCA

*HPRT1*_Rv GGTCCTTTTCACCAGCAAGCT

### Cell growth and viability assay

Cells were cultured in RPMI 1640 medium (Gibco) supplemented with 10% FBS. Cells were tested for mycoplasma and Short Tandem Repeats were characterized for authentication. One SS cell-line was used (SYO-1) [21]. Three LMS cell lines were included (JA192, LMS04 and LMS05). Quisinostat (Selleckchem) and trichostatin A (Selleckchem) were used for HDAC inhibition. Both compounds were dissolved in DMSO. Cells were seeded in triplicates on a 96-well plate and compounds were added after 24 hours. Cell viability was measured after 72 hours incubation with the compounds by adding PrestoBlue Cell Viability Reagent (Life Technologies) according to the manufacturers protocol. Fluorescence was measured reading the plate at 590 nm on a fluorometer (Victor3V, 1420 multi-label counter). Viability was determined in three independent experiments in triplicate.

### Connectivity map analysis

For Connectivity Map (CMAP) analysis the regulatory network was first determined using expression2kinase (maayanlab.net/X2K) based on the differentially expressed genes that were identified. Potential targeted therapies were identified based on the proteins in the regulatory network. The pipeline for identification of transcription factors and kinases is described in literature [22].

### Statistical software and figures

R statistical software (v3.4.4) was used for all statistical tests [13]. Network plots were generated with igraph (v1.2.1) R package and formatted with Cytoscape (v3.6.0) [23]. Chord diagrams were generated with GOplot (v1.0.2) [24]. All further graphs were generated with R package ggplot2 (v2.2.1). Cox regression was performed with the “coxph” function from the survival (v2.43) R package.

## Results

### Soft tissue sarcomas show different molecular profiles

Since soft tissue sarcomas are histologically classified according to their line of differentiation, we compared gene expression data from 206 soft tissue sarcoma samples in The Cancer Genome Atlas (TCGA) (Table 1) with normal tissues from the Genotype-Tissue Expression (GTEx) project. For this we used a deep neural network approach, enabling us to find similarities between normal tissues and tumors identified through hidden layers that would not be obvious in a direct comparison (such as a PCA analysis). First the TCGA and GTEx data were combined and normalized together (S1a Fig). Principal components were calculated for all samples, the principal components (9868 in total) for the GTEx data was used to train a neural network resulting in a prediction accuracy of 98% (S1b Fig). The neural network was then applied to the principal components from the TCGA sarcoma data.

**Table 1:** Soft tissue sarcoma subtypes in the TCGA.

abbreviation	histology	cases
DDLPS	dedifferentiated liposarcoma	50
MFS	myxofibrosarcoma	17
MPNST	malignant peripheral nerve sheath tumor	5
SS	synovial sarcoma	10
STLMS	leiomyosarcoma—soft tissue	53
ULMS	leiomyosarcoma—gynecologic	27
UPS	undifferentiated pleomorphic sarcoma	44

**Fig 1:**
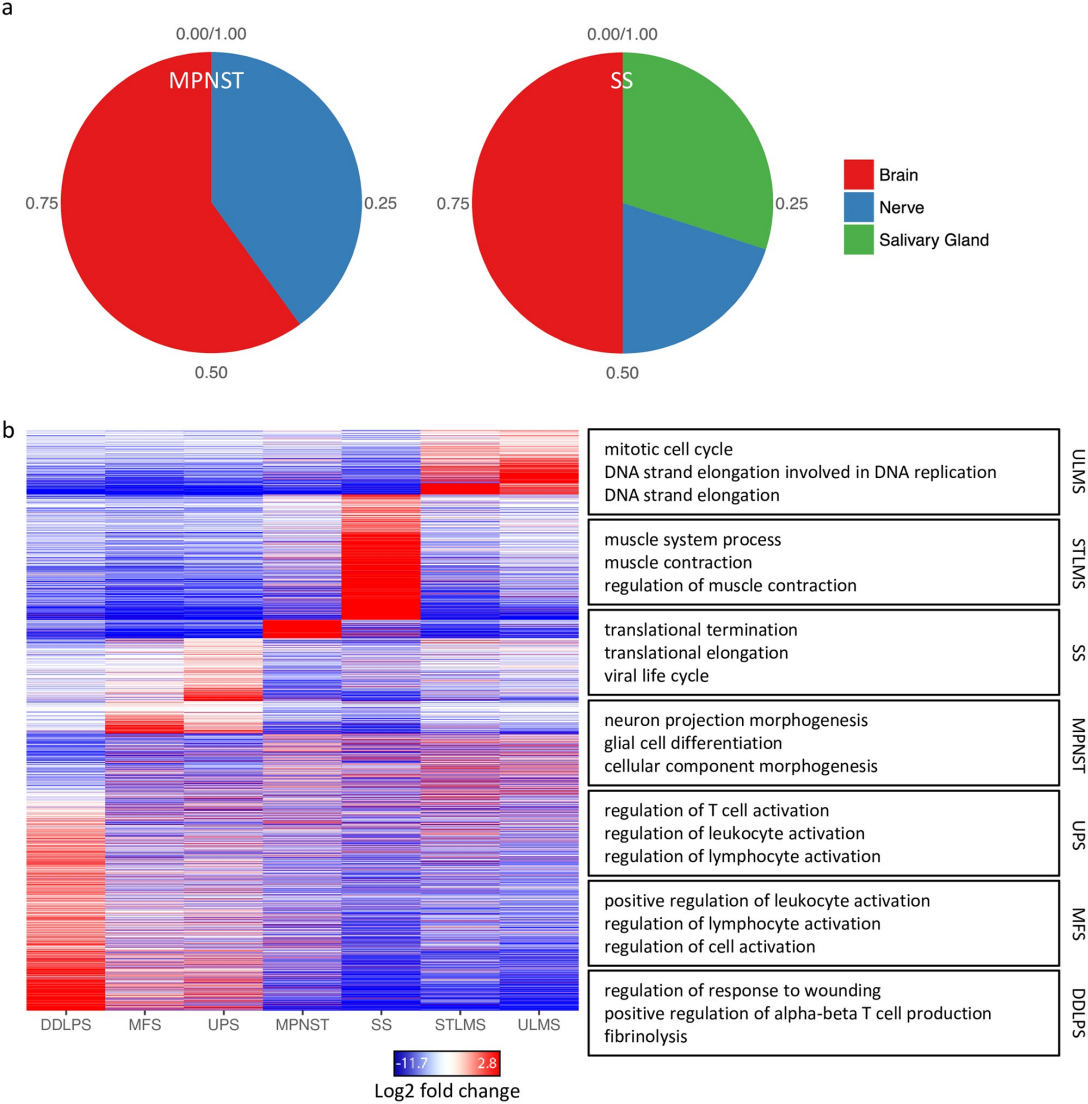
Relation to normal tissue and molecular profiles of soft tissue sarcomas. **(a)** A deep neural network was trained on GTEx expression data from normal tissue to investigate differentiation in the soft tissue sarcoma subtypes. MPNST and SS both showed the most specific differentiation and showed largest similarity with brain and nerve gene expression profiles. **(b)** Heat map plot of the identified signature genes in the different soft tissue sarcoma subtypes. The largest overlap in signature genes is seen between UPS and MFS (1201). Enriched GO terms in each of the signature genes are shown in the right panel. All GO terms have an adjusted P value lower then 1e-4.

As might be expected, ULMS was the only sarcoma subtype showing overlap with the expression patterns of normal uterus tissue as well as normal cervical tissue (S1c Fig). Moreover, STLMS was the only subtype showing similarity to blood vessel, which may be explained by the fact that a subset of STLMS are presumed to arise from small to medium sized veins [25]. However, both ULMS and STLMS also showed overlap with skin and brain tissue which is more difficult to understand at this point. Interestingly, we found large similarities between MPNST and SS, showing expression patterns very similar to tissue derived from the nervous system (brain and nerve). In addition SS showed some overlap with salivary gland which might be explained by the fact that 2 out of 10 SS were biphasic, of which the glandular epithelial elements may have caused the found similarity with salivary gland (Fig 1a). Surprisingly, MFS, and to a lesser extent UPS, showed a large overlap with normal adipose tissue. The overlap with adipose tissue in MFS and UPS is larger than found in DDLPS, which could be due to the selective sampling of DDLPS including the dedifferentiated component. For the other soft tissue sarcoma subtypes similarities were more dispersed since no specific normal tissue showed a large overlap with the tumor gene expression (S1c Fig).

To study the gene expression patterns of soft tissue sarcomas the TCGA expression data was normalized and differentially expressed genes (DEGs) were identified (Benjamini-Hochberg adjusted p value < 0.05 and logFC > 0) for all soft tissue sarcoma subtypes using Limma and Voom, comparing the subtypes to the other samples (S1d Fig). The number of DEGs per subtype ranged from 331 to 7784 (in STLMS and DDLPS respectively, 3156 DEGs on average) (S1e Fig). The DEGs were used to generate a heat map showing differences between soft tissue sarcoma subtypes. MFS and UPS showed the largest overlap in DEGs (1201 genes) followed by STLMS and ULMS (210 genes) (Fig 1b). Using EnrichR we tested for functional enrichment of the DEGs to identify GO terms associated with each of the subtypes. The DEGs from STLMS and MPNST showed a clear relation to differentiation; GO terms for STLMS related to muscle development and for MPNST the GO terms related to neuronal development. The top GO terms associated with ULMS were not related to muscle differentiation, but with cell cycle processes. However, significant GO terms associated with muscle differentiation were identified such as “muscle system process” (adjusted p = 6e-4) and “muscle contraction” (adjusted p = 3e-3) matching with the GO terms found in STLMS, which suggests that proliferation was more pronounced than differentiation in the ULMS compared to the STLMS samples. We did not identify GO terms related to differentiation for DDLPS, but, as can be seen in the heat map, we found that many of the identified GO terms associated with DDLPS, UPS and MFS overlapped. These included GO terms associated with the immune system which may reflect the presence of an inflammatory infiltrate in these tumors (Fig 1b).

### A random forest approach can differentiate between the soft tissue sarcoma subtypes

**Fig 2:**
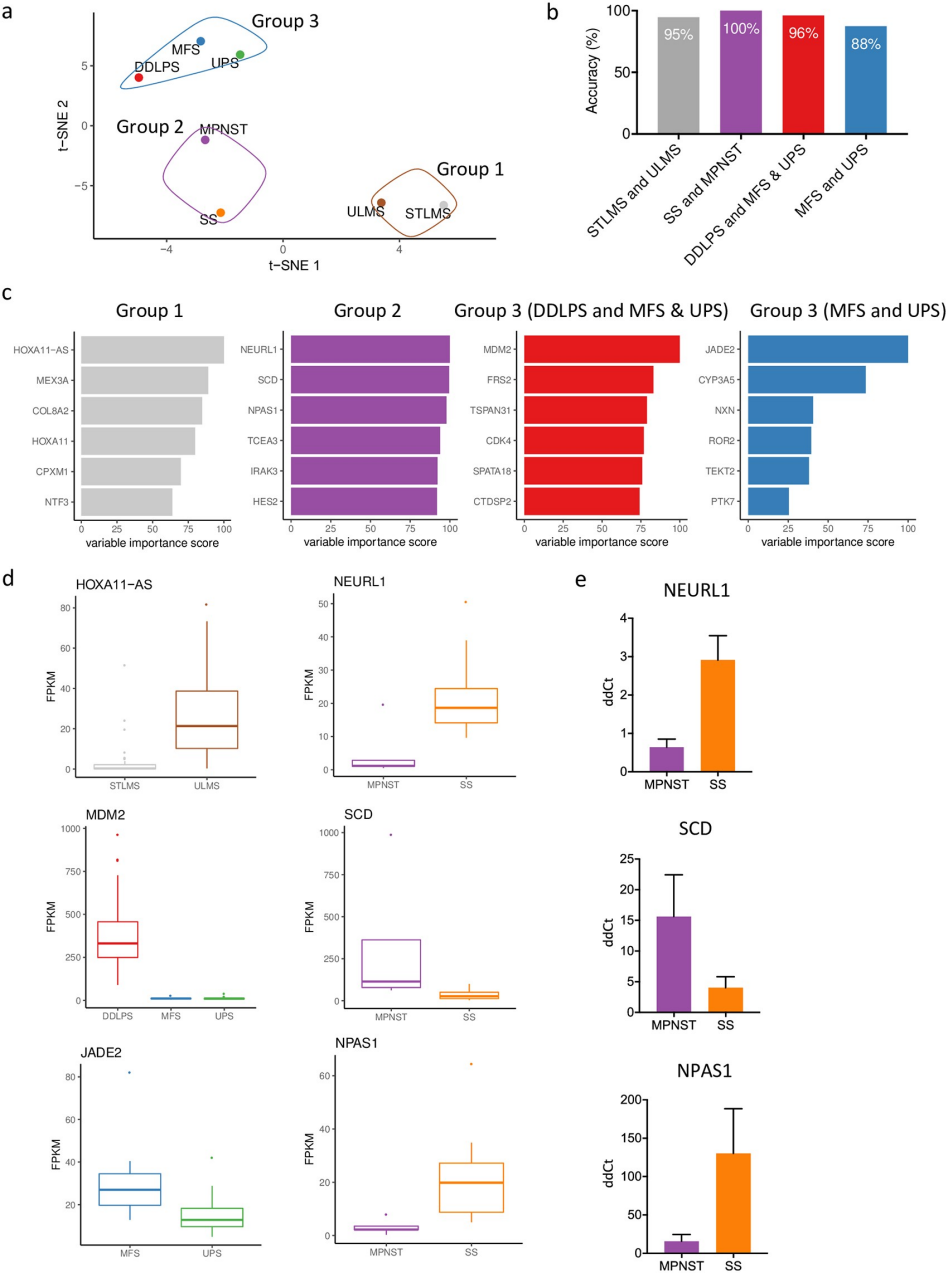
Diagnostic markers to distinguish within three subgroups. **(a)** T-SNE analysis of all soft tissue sarcoma subtypes in the TCGA. The first two components were used to generate the diagram. Three groups could be identified based on the molecular profile: group 1 (STLMS and ULMS); group 2 (SS and MPNST); group 3 (DDLPS, UPS and MFS). **(b)** A machine learning random forest analysis was trained and tested on a test dataset. Random forests were generated to differentiate between STLMS and ULMS, SS and MPNST, DDLPS and MFS with UPS and last between MFS and UPS. Within the three identified groups a prediction accuracy of over 95% was reached, except when differentiating between UPS and MFS (88%). **(c)** From the random forest models, the top five genes were selected based on their Gini index, score is shown relative to the best diagnostic marker. **(d)** Gene expression (in FPKM) for the best subtype predictor within the identified groups is shown in the boxplots on the left. On the right the top three subtype predictors are shown for group 2 (MPNST and SS), which were verified using qRT-PCR. The box shows the interquartile range from Q1 to Q3 and the mean. The whiskers show the highest and lowest values. Suspected outliers (interquartile range * 1.5) are shown as separate dots. **(e)** qRT-PCR validation in independent cohort: Delta-delta Ct (ddCt) values are shown for the top three diagnostic genes identified for group 2 (MPNST and SS). Expression pattern is similar to what was found in the TCGA data. Expression was normalized with a housekeeping gene (*HPRT1*).

To investigate the similarities of the molecular profiles of the different soft tissue sarcoma subtypes we performed a t-SNE analysis on the expression data (S2a Fig). The average of the first two components for the different subtypes is shown in Fig 2a. In the t-SNE analysis, three clusters of soft tissue sarcoma subtypes were identified. MFS, UPS and DDLPS clustered together, in line with the undifferentiated sometimes pleomorphic morphology of these tumors. ULMS and STLMS also cluster together. The third cluster consisted of MPNST and SS, for which distinction based on morphology alone is often impossible.

As a deep neural network is not informative on the biological differences between these subtypes, we therefore used a random forest machine learning approach to identify subtype defining genes. The samples were divided into test and training groups at random. The resulting random forest reached a subtype prediction accuracy of over 95% for all groups, except in differentiating between MFS and UPS (where it reached an accuracy of 88%) (Fig 2b).

Differentially expressed genes (adjusted p<0.05) were used to generate the random forest. Important genes were identified based on their variable importance index (Fig 2c). Top genes in group 1 (STLMS and ULMS) included *HOXA11* and its anti-sense RNA (*HOXA11-AS*) were identified. *HOXA11* and *HOXA11-AS* have both been described to be important regulators of uterine development and homeostasis [26]. For group 2 (MPNST and SS) genes related to neural differentiation such as *NEURL1* and *NPAS1* were identified, which were found to be upregulated in synovial sarcomas, while SCD, an enzyme involved in fatty acid biosynthesis, is more highly expressed in MPNST. For the third group (DDLPS, UPS and MFS), we first compared DDLPS with the UPS and MFS together. As previously described and already widely implemented in routine diagnostics, expression of *MDM2* and *CDK4* (which is part of the 12q13-15 amplification characteristic of DDLPS) were identified as diagnostic markers to identify DDLPS [27]. *FRS2*, *TSPAN31* and *CTDSP2* are located near the amplified *MDM2* on chromosome 12 and therefore most likely also part of the same amplified region that characterizes DDLPS. In Fig 2d, we visualized gene expression levels of the genes with the highest variable importance scores for each of the four comparisons. *JADE2* showed the highest variable importance score for the differentiation between UPS and MFS although expression still somewhat overlapped, confirming the large molecular and morphological similarity between the two entities (Fig 2d).

To verify the diagnostic markers that were identified for group 2 (MPNST and SS) using the random forest algorithm we used qRT-PCR on an independent cohort of nine samples. Indeed, the expression patterns of *NEURL1*, *SCD* and *NPAS1* were similar in the independent cohort (Fig 2e).

### Soft tissue sarcoma subtypes have distinct prognostic genes

We identified prognostic genes for all annotated soft tissue sarcoma subtypes, except MPNST (with only five samples available). First, the optimal gene expression cutoff was calculated for all the 24168 genes that met the defined thresholds in the TCGA soft tissue sarcoma expression data. Next, disease-free interval (DFI) (time to local recurrence or distant metastases) was tested using the Hothorn and Lausen statistical test; DFI was used as the read-out.

**Fig 3:**
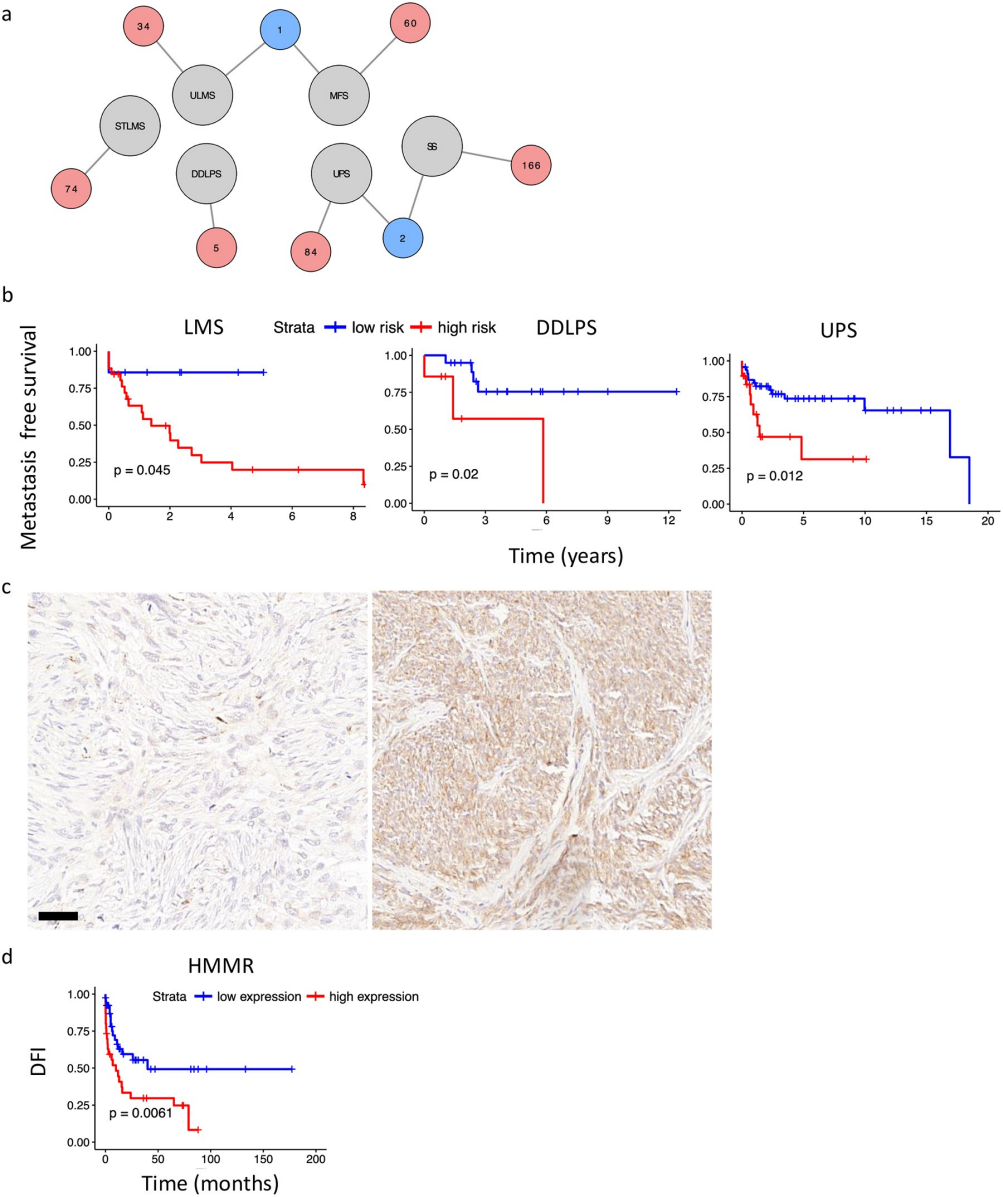
Novel prognostic biomarkers in soft tissue sarcomas. **(a)** All identified prognostic genes and their overlap within the different soft tissue sarcoma subtypes is shown with a network diagram. UPS and SS share two prognostic genes and ULMS and MFS share one. Furthermore, all identified genes were specific for each sarcoma subtype. Number of prognostic genes are shown in the red circles, tumor types in the gray circles and number of overlapping prognostic genes in the blue circles. **(b)** The k-Nearest Neighbor algorithm was also used with expression data for the strongest prognostic genes identified in both the French Sarcoma Group and TCGA expression data. The algorithm was trained on the first and tested on the second cohort. Both were found to be significant predictors of the metastasis-free interval. **(c)** HMMR protein expression was tested using IHC on a LMS TMA. The left panel shows a representative sample with low expression, on the right a sample with high HMMR expression. Scale bar indicates 50 μm. **(d)** High HMMR protein expression as seen in an independent cohort of LMS from our archives is associated with poor outcome.

In total 429 genes were found to be strong predictors (favorable or unfavorable) of DFI (p < 0.001) (S3 Table). Most genes were identified for SS (166 genes) while 74 and 34 genes were identified for STLMS and ULMS respectively. Interestingly, there was very little overlap between the prognostic genes for the different subtypes. Two overlapping prognostic genes (*KLF6* and *MT1F*) were found for UPS and SS and one (*NPM2*) for ULMS and MFS. No overlapping prognostic genes were found between STLMS and ULMS (Fig 3a). Furthermore, only one gene (*CDCA3* identified in STLMS) was found to overlap between the 67 described CINSARC genes and the soft tissue sarcoma subtype specific prognostic genes identified in the current study. From the 429 identified prognostic genes 201 were new, 228 had however been previously identified in other (non-sarcoma) tumor types in the Protein Atlas database (S3a Fig).

To cross-check the identified prognostic genes identified for LMS, DDLPS and UPS, we used expression data from the French Sarcoma Group [9]. The French Sarcoma Group array data was first normalized (S3b Fig). The data contained information on the metastasis-free interval but not DFI as was used by us for the TCGA data. The French Sarcoma Group data was split in two groups. Genes that were significant prognostic genes for DFI in the TCGA and the metastasis-free interval in the first French Sarcoma Group cohort (both with p < 0.05) were considered for further analysis (S4 Table). From the identified genes, strong prognostic genes were used in a k-nearest neighbor analysis. For LMS *HMMR*, *MXD4* and *BRCA2* were identified, for DDLPS *KLF6* was found to be a strong prognostic gene while for UPS *PCMTD2*, *TNXA*, *TMEM65*, *SNRNP48* were identified. The k-nearest neighbor algorithm was trained on the first group and tested on the second group in the French Sarcoma samples. The k-nearest neighbor algorithm was a significant predictor for the metastasis-free interval for LMS, DDLPS and UPS in the second group (p = 0.045, p = 0.02 and p = 0.012 respectively) (Fig 3b), outperforming the reported CINSARC classification in the second cohort (LMS p = 0.24, DDLPS p = 0.14 and UPS p = 0.038) (S3c Fig).

HMMR was identified as a significant (p<0.05) prognostic gene for DFI and the metastasis-free interval in LMS. In an independent validation set of 70 LMS cases, we verified using immunohistochemistry with automated scoring that high protein expression of HMMR was associated with a shorter DFI (p = 0.0061) (Fig 3c & 3d). For the second cohort, manual scoring was compared with automated scoring and results were similar. Prognostic value of HMMR was further compared to the FNCLCC grading system. In a multivariate Cox-regression it was found that the HMMR staining (p = 0.0039) retained significance and was a better predictor than FNCLCC histological grade (p = 0.285).

### Systems analysis of the soft tissue sarcoma subtype-specific genes identify targeted therapies

**Fig 4:**
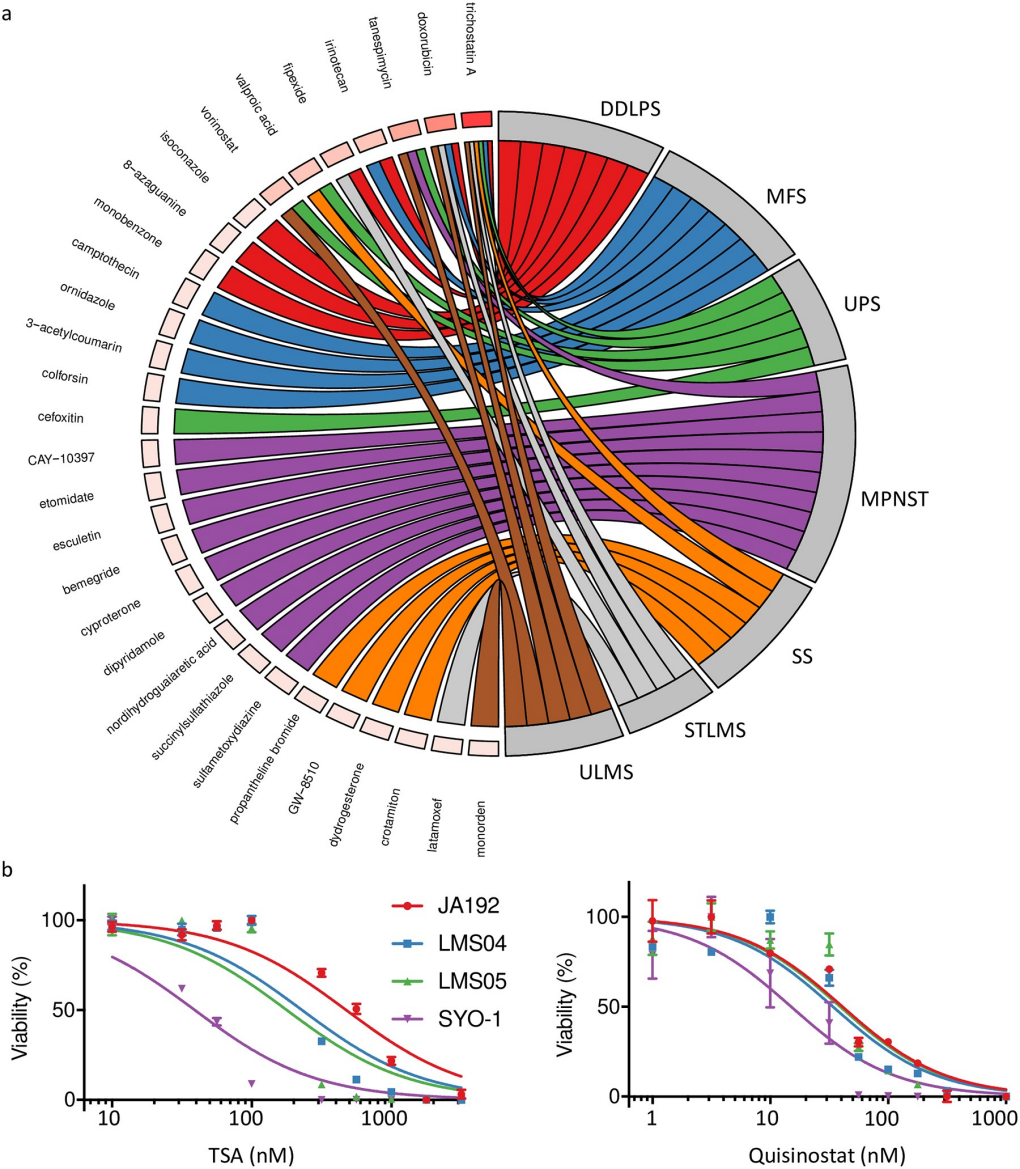
CMAP analysis to identify novel therapies. **(a)** CMAP analysis identifies potential drugs based on the expression profile. The chord diagram shows links between the drugs and soft tissue sarcoma subtypes. Some compounds such as trichostatin A, doxorubicin and tanespimycin show connections with multiple soft tissue sarcoma subtypes, which is illustrated by the box color for each drug (darker red indicates more connections). **(b)** The dose response curves are shown for both trichostatin A (TSA) and quisinostat as tested in one SS (SYO-1) and three LMS (JA192, LMS04 and LMS05) cell-lines.

To identify novel targeted therapies gene expression data was used to infer the regulatory transcription factors and kinases in the different soft tissue sarcoma subtypes. First, the signature genes for each soft tissue sarcoma subtype were used to infer the transcription factors that were most likely to regulate those genes based on data from the ChIP-seq Enrichment Analysis (ChEA) database [22]. The most important kinases regulating these transcription factors were inferred using the Kinase Enrichment Analysis [22]. Based on the identified transcription factors and kinases, tumor subtype specific drugs were identified based on the Connectivity Map (CMAP) drug data (with kinases and transcription factors as input). Doxorubicin, which is commonly used as systemic treatment for STS, was identified as a potentially effective therapy for most soft tissue sarcoma subtypes, validating our analysis approach. Trichostatin A, a HDAC inhibitor, was predicted to be potentially efficient in all soft tissue sarcoma subtypes, while another HDAC inhibitor, Vorinostat, was identified for UPS and ULMS. Tanespimycin was identified for UPS, ULMS and MPNST, which is an inhibitor of Hsp90 and currently used in clinical trials for solid tumors (Fig 4a and S5 Table).

While sensitivity to HDAC inhibition is known for translocation driven tumors like synovial sarcoma [28], for LMS this has not been extensively studied. We thus performed cell viability assays on three LMS cell lines (JA192, LMS04 and LMS05), treated with two HDAC inhibitors (quisinostat and trichostatin A), with one SS cell line (SYO-1) as positive control (Fig 4b). For both compounds the half maximal inhibitory concentration (IC50) was determined. For trichostatin A (TSA) an IC50 ranging from 39 to 474 nM was found (JA192: 474 nM; LMS04: 229 nM; LMS05: 178 nM; SYO-1: 39 nM). Although all cell-lines were sensitive to TSA, SYO-1 was more sensitive compared to the LMS cells. However, for quisinostat a low IC50 was found for all cell-lines; between 15 and 41 nM (JA192: 41 nM, LMS04: 34 nM; LMS05: 39 nM; SYO-1: 15 nM). These results indicate that LMS and SS cell lines are highly sensitive to HDAC inhibition by quisinostat.

## Discussion

Accurate diagnosis and prediction of biological behavior is a challenge for soft tissue sarcoma pathologists. These tumors are rare and often overlap in their morphology, while subtype specific diagnostic and prognostic markers are scarce. As an increasing amount of transcriptome sequencing data becomes available, even for rare cancers such as soft tissue sarcomas, new methods need to be developed to identify novel diagnostic and prognostic biomarkers for these tumors from existing data.

Here we used machine learning algorithms to identify similarities and differences between soft tissue sarcoma subtypes and normal human tissue from the GTEx data.

Using a deep neural network, we demonstrate that SS and MPNST mostly correspond to neural related tissues. MPNST often arises from or within nerves; therefore, it is likely a tumor originating from neural related tissue, while for synovial sarcoma the cell of origin and line of differentiation have been unclear. Our observation of the neural related tissue as a potential tissue of origin confirms previous suggestions [29]. The deep neural network also identified that cervix and uterine tissue showed the largest overlap with ULMS as is expected. Other findings however illustrate the limitations in comparing gene expression of normal tissue with tumor, such as the large overlap in gene expression between skin and adrenal gland with ULMS or the large overlap found between SS and salivary gland (that could be explained due to the biphasic SS samples displaying epithelial elements). These findings in part could be explained by the fact that the sequencing is performed on tissue containing many different cell types, including immune and stromal cells. Single cell sequencing and projects such as the Human Cell Atlas [30] could in the future shed more light on the tissue of origin for soft tissue sarcomas.

Using a random forest analysis, we identified subtype specific genes that can be used as diagnostic markers within the three groups of soft tissue sarcoma subtypes that were identified based on their molecular profile and morphology. For instance, *NEURL1* was one of the genes highly expressed in SS as compared to MPNST. *NEURL1* is an important determinant of neural tissue differentiation and functions as a tumor suppressor which is inactivated during malignant progression of astrocytic tumors [31]. In line with this, the lower expression of *NEURL1* could be explained by recurring losses of chromosome 10 in 48% of MPNST [32]. *SCD* was found to be highly expressed in MPNST compared to SS. *SCD* has been found to associate with a poor prognosis in breast and lung cancer. Moreover, *SCD* can be directly inhibited with the small molecule MF-438 which sensitized adenocarcinoma cells to cisplatin treatment [33,34]. It was previously found that when SS was treated with a HDAC inhibitor, neural differentiation was induced [28]. Furthermore, treatment with BMP4 or FGF2 restored expression of neural tissue related genes in SS [35]. Our study further confirms neural differentiation in SS, as shown using hidden layers in a deep neural network. Future validation studies should indicate whether the diagnostic biomarkers that we identified here can also be used immunohistochemically in the differential diagnosis.

We identified subtype specific prognostic genes using Kaplan-Meier analysis on all individual genes combined with a k-nearest neighbor algorithm to accurately predict the disease-free interval (DFI). DFI was previously shown to be one of the strongest outcome measurements for soft tissue sarcomas [15]. For all genes the cut off was determined first and the DFI for high and low expression was calculated. This Kaplan-Meier approach was previously used on 17 other cancer types, not including soft tissue sarcomas [11]. Although this method results in tumor subtype specific prognostic genes that can predict outcome, a major challenge is to correct for multiple testing. Here we used an independent cohort from the French Sarcoma Group to validate strong prognostic genes for LMS, DDLPS and UPS. However, for this independent cohort only data on metastasis were available, whereas the TCGA also contained data on loco-regional recurrence. Using both data sets, overlapping prognostic genes were identified which could be considered strong prognostic genes. For the other tumor subtypes, to our knowledge, there are no available expression data sets with accurate follow up data to perform cross-validation. Interestingly we found only one gene, *CDCA3*, overlapped between the prognostic genes we identified in the TCGA soft tissue sarcoma data and the CINSARC prognosticator. We likely did not identify a larger overlap because the CINSARC study aimed to identify a general prognosticator for soft tissue sarcomas, which is not subtype specific. In addition, the outcome used was different; we used DFI as an outcome measurement while in the CINSARC study metastasis was used. Moreover, we identified subtype specific prognostic genes using a Kaplan-Meier approach which does not only take outcome but also time to events into account. Here we showed that subtype specific prognostic genes outperformed general prognostic genes.

For one of the identified genes, HMMR, we confirmed that high protein expression was associated with poor outcome of LMS. Further we confirmed that HMMR expression outperformed the FNCLCC histological grading to predict outcome. Recently it was shown that LMS displays hallmarks of “BRCAness” through identification of mutation signatures and alterations in genes related to homologous recombination [36]. Here we identified strong prognostic genes for LMS, two of which were related to homologous repair (*BRCA2* and *HMMR*). HMMR forms a complex with BRCA1 or BRCA2 together with other proteins, and high expression of *HMMR* was associated with poor survival in liver, pancreatic and lung cancer [11]. Possibly, defects in the homologous repair pathway could result in over-expression of *HMMR* in an attempt to compensate for other defective proteins. The involvement of genes related to “BRCAness” and to disease outcome warrants further studies.

A regulatory network reconstruction combined with the CMAP drug data revealed not only the commonly used drug doxorubicin, but also indicated that HDAC inhibitors could be a potential treatment for many different soft tissue sarcoma subtypes. Recent studies indeed suggest that HDAC inhibitors may be effective in treating soft tissue sarcomas. In liposarcoma it was shown that HDAC inhibitors increase apoptosis and anti-proliferation effects [37]. In SS HDAC inhibitors cause disruption of the SS18-SSX oncoprotein resulting in apoptosis [28]. Another study found HDAC inhibitors lead to apoptosis in SS cell-lines [38]. In other sarcoma subtypes HDAC inhibitors have not been studied extensively. One uterine LMS cell line was tested and shown to be sensitive to the pan HDAC inhibitor ITF2357 with a synergistic effect when combined with doxorubicin [39]. In this study we further investigated LMS sensitivity to HDAC inhibition using quisinostat and trichostatin A. We included three LMS cell-lines, one ULMS (LMS04) and two STLMS (LMS05 and JA192). As SS was previously found to be sensitive to HDAC inhibition we also included one SS cell-line (SYO-1) as a positive control. SS showed a greater sensitivity to TSA, however, quisinostat showed a very low IC50 (15–41 nM) in all cell lines. Thus, quisinostat might be further explored as a potential therapy for both ULMS and STLMS.

In conclusion, three groups of soft tissue sarcoma subtypes included in the TCGA study were identified based on similarities in their expression profiles, corresponding to their overlapping morphology. Using a random forest analysis, novel diagnostic markers were identified that may distinguish between soft tissue sarcoma subtypes within these three groups, including *NEURL1* that was highly expressed in SS as compared to MPNST. Next, using a Kaplan-Meier analysis, prognostic genes were identified. Of these, HMMR protein expression was confirmed to be associated with poor outcome in an independent cohort of LMS from our archives. A network reconstruction combined with CMAP data revealed that HDAC inhibitors could be effective therapy in different soft tissue sarcoma subtypes, which we confirmed in LMS and SS cell-lines.

In conclusion, machine learning algorithms uncovered diagnostic biomarkers, prognostic genes and identified potential novel therapeutic targets for soft tissue sarcomas. This study thereby illustrates the power of different machine learning algorithms to improve our understanding of rare cancers using existing datasets.

## Supporting information

S1 Fig Relation between normal tissue and molecular profiles of soft tissue sarcomas. **(a)** Combined and normalized expression data from the GTEx (red) and TCGA (blue), showing 100 samples from both data sets. **(b)** Deep neural network training on GTEx principal component data resulted in a prediction accuracy of 98% in 30 epochs. Loss and accuracy are shown over the 30 training epochs. **(c)** Overlap of the different types of soft tissue sarcomas with normal tissue from the GTEx. **(d)** Normalized expression from TCGA soft tissue sarcoma samples. **(e)** Differentially expressed genes for all soft tissue sarcoma subtypes in the TCGA, compared to the other soft tissue sarcoma subtypes.
(TIF)

S2 Fig Diagnostic markers to distinguish within three subgroups. **(a)** t-SNE analysis of all soft tissue sarcoma samples, colored according to the subtype.
(TIF)

S3 Fig Novel prognostic biomarkers in soft tissue sarcomas. **(a)** Differences and overlap with the genes that are prognostic, as found in the Pathology Atlas analysis. Many of the identified prognostic genes are also prognostic genes in other cancer types. Number of prognostic genes are shown in the red circles, tumor types in the gray circles and all tumor types analyzed in the protein atlas are shown as a collection in the blue circle. **(b)** Normalized expression data from the French Sarcoma Group array expression data from sarcomas. **(c)** Classification according to the CINSARC C1 or C2 classification in the second cohort.
(TIF)

S1 Table Tissue types present in the GTEx data. (XLSX)

S2 Table Clinicopathological details for the newly constructed TMA. (XLSX)

S3 Table Strong predictors of the DFI. (XLSX)

S4 Table Significant prognostic genes in both the TCGA and French Sarcoma Group. (XLSX)

S5 Table Subtype specific drugs identified from the CMAP data. (XLSX)

## References

[1] Taylor BS, Barretina J, Maki RG, Antonescu CR, Singer S, Ladanyi M. Advances in sarcoma genomics and new therapeutic targets. Nat Rev Cancer. 2011/07/15. 2011;11: 541–557. 10.1038/nrc3087 21753790 PMC3361898

[2] Fletcher CDM, Bridge JA, Hogendoorn PCW, Mertens F. WHO Classification of Tumours of Soft Tissue and Bone. 2013.

[3] Abeshouse A, Adebamowo C, Adebamowo SN, Akbani R, Akeredolu T, Ally A, et al Comprehensive and Integrated Genomic Characterization of Adult Soft Tissue Sarcomas. Cell. 2017/11/04. 2017;171: 950–965.e28. 10.1016/j.cell.2017.10.014 29100075 PMC5693358

[4] Van Der Maaten L, Hinton G. Visualizing Data using t-SNE [Internet]. Journal of Machine Learning Research. 2008. http://www.jmlr.org/papers/volume9/vandermaaten08a/vandermaaten08a.pdf

[5] Sorlie T, Perou CM, Tibshirani R, Aas T, Geisler S, Johnsen H, et al Gene expression patterns of breast carcinomas distinguish tumor subclasses with clinical implications. Proc Natl Acad Sci. 2001/09/13. 2001;98: 10869–10874. 10.1073/pnas.191367098 11553815 PMC58566

[6] Lu J, Getz G, Miska EA, Alvarez-Saavedra E, Lamb J, Peck D, et al MicroRNA expression profiles classify human cancers. Nature. Nature Publishing Group; 2005;435: 834 10.1038/nature03702 15944708

[7] Röhrich M, Koelsche C, Schrimpf D, Capper D, Sahm F, Kratz A, et al Methylation-based classification of benign and malignant peripheral nerve sheath tumors. Acta Neuropathol. 2016/02/10. 2016;131: 877–887. 10.1007/s00401-016-1540-6 26857854

[8] Capper D, Jones DTW, Sill M, Hovestadt V, Schrimpf D, Sturm D, et al DNA methylation-based classification of central nervous system tumours. Nature. 2018/03/15. 2018;555: 469–474. 10.1038/nature26000 29539639 PMC6093218

[9] Chibon F, Lagarde P, Salas S, Perot G, Brouste V, Tirode F, et al Validated prediction of clinical outcome in sarcomas and multiple types of cancer on the basis of a gene expression signature related to genome complexity. Nat Med. 2010/06/29. 2010;16: 781–787. 10.1038/nm.2174 20581836

[10] Lesluyes T, Delespaul L, Coindre JM, Chibon F. The CINSARC signature as a prognostic marker for clinical outcome in multiple neoplasms. Sci Rep. 2017/07/16. 2017;7: 5480 10.1038/s41598-017-05726-x 28710396 PMC5511191

[11] Uhlen M, Zhang C, Lee S, Sjostedt E, Fagerberg L, Bidkhori G, et al A pathology atlas of the human cancer transcriptome. Science (80-). 2017/08/19. 2017;357 10.1126/science.aan2507 28818916

[12] The Genotype-Tissue Expression (GTEx) project. Nat Genet. 2013;45: 580–585. 10.1038/ng.2653 23715323 PMC4010069

[13] null null. R: A Language and Environment for Statistical Computing [Internet]. Vienna, Austria; 2017.

[14] Robinson MD, Oshlack A. A scaling normalization method for differential expression analysis of RNA-seq data. Genome Biol. BioMed Central; 2010;11 10.1186/gb-2010-11-3-r25 20196867 PMC2864565

[15] Liu J, Lichtenberg T, Hoadley KA, Poisson LM, Lazar AJ, Cherniack AD, et al An Integrated TCGA Pan-Cancer Clinical Data Resource to Drive High-Quality Survival Outcome Analytics. Cell. 2018/04/07. 2018;173: 400–416.e11. 10.1016/j.cell.2018.02.052 29625055 PMC6066282

[16] Lausen B, Hothorn T, Bretz F, Schumacher M. Assessment of optimal selected prognostic factors. Biometrical J. WILEY-VCH Verlag; 2004;46: 364–374. 10.1002/bimj.200310030

[17] De Graaff MA, Cleton-Jansen AM, Szuhai K, Bovée JVMG. Mediator complex subunit 12 exon 2 mutation analysis in different subtypes of smooth muscle tumors confirms genetic heterogeneity. Hum Pathol. 2013/03/23. 2013;44: 1597–1604. 10.1016/j.humpath.2013.01.006 23517922

[18] Baranski Z, Booij TH, Cleton-Jansen AM, Price LS, van de Water B, Bovee J V, et al Aven-mediated checkpoint kinase control regulates proliferation and resistance to chemotherapy in conventional osteosarcoma. J Pathol. 2015/03/11. 2015;236: 348–359. 10.1002/path.4528 25757065

[19] Cleven AH, Sannaa GA, Briaire-de Bruijn I, Ingram DR, van de Rijn M, Rubin BP, et al Loss of H3K27 tri-methylation is a diagnostic marker for malignant peripheral nerve sheath tumors and an indicator for an inferior survival. Mod Pathol. 2016/03/19. 2016;29: 582–590. 10.1038/modpathol.2016.45 26990975 PMC4948583

[20] Prieto-Granada CN, Wiesner T, Messina JL, Jungbluth AA, Chi P, Antonescu CR. Loss of H3K27me3 Expression Is a Highly Sensitive Marker for Sporadic and Radiation-induced MPNST. Am J Surg Pathol. 2015/12/10. 2016;40: 479–489. 10.1097/PAS.0000000000000564 26645727 PMC4882106

[21] Kawai A, Naito N, Yoshida A, Morimoto Y, Ouchida M, Shimizu K, et al Establishment and characterization of a biphasic synovial sarcoma cell line, SYO-1. Cancer Lett. 2004/01/28. 2004;204: 105–113. 10.1016/j.canlet.2003.09.031 14744540

[22] Chen EY, Xu H, Gordonov S, Lim MP, Perkins MH, Ma’ayan A. Expression2Kinases: mRNA profiling linked to multiple upstream regulatory layers. Bioinformatics. 2011/11/15. 2012;28: 105–111. 10.1093/bioinformatics/btr625 22080467 PMC3244772

[23] Shannon P, Markiel A, Ozier O, Baliga NS, Wang JT, Ramage D, et al Cytoscape: a software environment for integrated models of biomolecular interaction networks. Genome Res. 2003/11/05. 2003;13: 2498–2504. 10.1101/gr.1239303 14597658 PMC403769

[24] Walter W, Sanchez-Cabo F, Ricote M. GOplot: an R package for visually combining expression data with functional analysis. Bioinformatics. 2015/05/13. 2015;31: 2912–2914. 10.1093/bioinformatics/btv300 25964631

[25] Lazar A, Evans H, Shipley J. Smooth-muscle tumors. Leiomyoma of deep soft tissue Leiomyosarcoma. WHO classification of tumours of soft tissue and bone. Lyon: IARC; 2013 p. 111–3.

[26] Chau YM, Pando S, Taylor HS. HOXA11 silencing and endogenous HOXA11 antisense ribonucleic acid in the uterine endometrium. J Clin Endocrinol Metab. 2002/06/07. 2002;87: 2674–2680. 10.1210/jcem.87.6.8527 12050232

[27] Binh MB, Sastre-Garau X, Guillou L, de Pinieux G, Terrier P, Lagace R, et al MDM2 and CDK4 immunostainings are useful adjuncts in diagnosing well-differentiated and dedifferentiated liposarcoma subtypes: a comparative analysis of 559 soft tissue neoplasms with genetic data. Am J Surg Pathol. 2005/09/15. 2005;29: 1340–1347. 10.1097/01.pas.0000170343.09562.39 16160477

[28] Laporte AN, Poulin NM, Barrott JJ, Wang XQ, Lorzadeh A, Vander Werff R, et al Death by HDAC Inhibition in Synovial Sarcoma Cells. Mol Cancer Ther. 2017/09/08. 2017;16: 2656–2667. 10.1158/1535-7163.MCT-17-0397 28878027

[29] Ishibe T, Nakayama T, Aoyama T, Nakamura T, Toguchida J. Neuronal differentiation of synovial sarcoma and its therapeutic application. Clin Orthop Relat Res. 2008/06/20. 2008;466: 2147–2155. 10.1007/s11999-008-0343-z 18563503 PMC2493002

[30] Regev A, Teichmann SA, Lander ES, Amit I, Benoist C, Birney E, et al The human cell atlas. Elife. 2017;6 10.7554/eLife.27041 29206104 PMC5762154

[31] Nakamura H, Yoshida M, Tsuiki H, Ito K, Ueno M, Nakao M, et al Identification of a human homolog of the Drosophila neuralized gene within the 10q25.1 malignant astrocytoma deletion region. Oncogene. 1998/03/31. 1998;16: 1009–1019. 10.1038/sj.onc.1201618 9519875

[32] Bridge RS, Bridge JA, Neff JR, Naumann S, Althof P, Bruch LA. Recurrent chromosomal imbalances and structurally abnormal breakpoints within complex karyotypes of malignant peripheral nerve sheath tumour and malignant triton tumour: a cytogenetic and molecular cytogenetic study. J Clin Pathol. 2004 pp. 1172–1178. 10.1136/jcp.2004.019026 15509679 PMC1770473

[33] Holder AM, Gonzalez-Angulo AM, Chen H, Akcakanat A, Do KA, Symmans WF, et al High stearoyl-CoA desaturase 1 expression is associated with shorter survival in breast cancer patients. Breast Cancer Res Treat. 2013;137: 319–327. 10.1007/s10549-012-2354-4 23208590 PMC3556743

[34] Pisanu ME, Noto A, De Vitis C, Morrone S, Scognamiglio G, Botti G, et al Blockade of Stearoyl-CoA-desaturase 1 activity reverts resistance to cisplatin in lung cancer stem cells. Cancer Lett. 2017/08/12. 2017;406: 93–104. 10.1016/j.canlet.2017.07.027 28797843

[35] Ishibe T, Nakayama T, Aoyama T, Nakamura T, Toguchida J. Neuronal Differentiation of Synovial Sarcoma and Its Therapeutic Application. Clin Orthop Relat Res. 2008 pp. 2147–2155. 10.1007/s11999-008-0343-z 18563503 PMC2493002

[36] Chudasama P, Mughal SS, Sanders MA, Hübschmann D, Chung I, Deeg KI, et al Integrative genomic and transcriptomic analysis of leiomyosarcoma. Nat Commun. 2018/01/13. 2018;9: 144 10.1038/s41467-017-02602-0 29321523 PMC5762758

[37] Ou W-B, Zhu J, Eilers G, Li X, Kuang Y, Liu L, et al HDACi inhibits liposarcoma via targeting of the MDM2-p53 signaling axis and PTEN, irrespective of p53 mutational status. Oncotarget. 2015/04/19. 2015;6: 10510–20. 10.18632/oncotarget.3230 25888633 PMC4496371

[38] Bernhart E, Stuendl N, Kaltenegger H, Windpassinger C, Donohue N, Leithner A, et al Histone deacetylase inhibitors vorinostat and panobinostat induce G1 cell cycle arrest and apoptosis in multidrug resistant sarcoma cell lines. Oncotarget. 2017/11/05. 2017;8: 77254–77267. 10.18632/oncotarget.20460 29100385 PMC5652778

[39] Di Martile M, Desideri M, Tupone MG, Buglioni S, Antoniani B, Mastroiorio C, et al Histone deacetylase inhibitor ITF2357 leads to apoptosis and enhances doxorubicin cytotoxicity in preclinical models of human sarcoma. Oncogenesis. 2018/02/24. 2018;7: 20 10.1038/s41389-018-0026-x 29472530 PMC5833676

