# Supplementary figures and images for: Machine learning analysis of gene expression data reveals novel diagnostic and prognostic biomarkers and identifies therapeutic targets for soft tissue sarcomas
Source: PLoS Comput Biol. 2019 Feb 20;15(2):e1006826. doi: 10.1371/journal.pcbi.1006826 (PMC6398862; doi:10.1371/journal.pcbi.1006826)

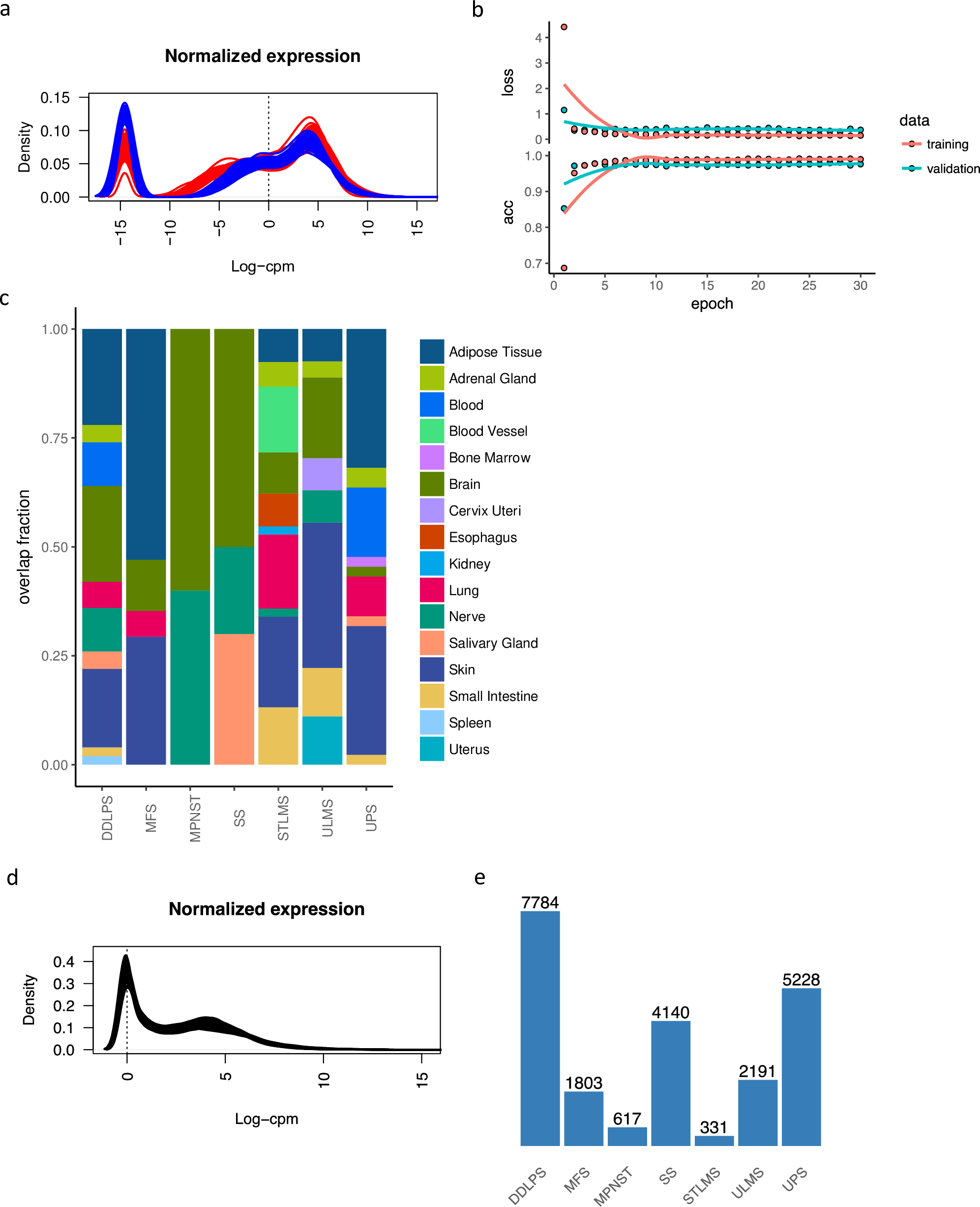

Supplement: S1 Fig — (a) Combined and normalized expression data from the GTEx (red) and TCGA (blue), showing 100 samples from both data sets. (b) Deep neural network training on GTEx principal component data resulted in a prediction accuracy of 98% in 30 epochs. Loss and accuracy are shown over the 30 training epochs. (c) Overlap of the different types of soft tissue sarcomas with normal tissue from the GTEx. (d) Normalized expression from TCGA soft tissue sarcoma samples. (e) Differentially expressed genes for all soft tissue sarcoma subtypes in the TCGA, compared to the other soft tissue sarcoma subtypes. (TIF) [file pcbi.1006826.s001.tif]

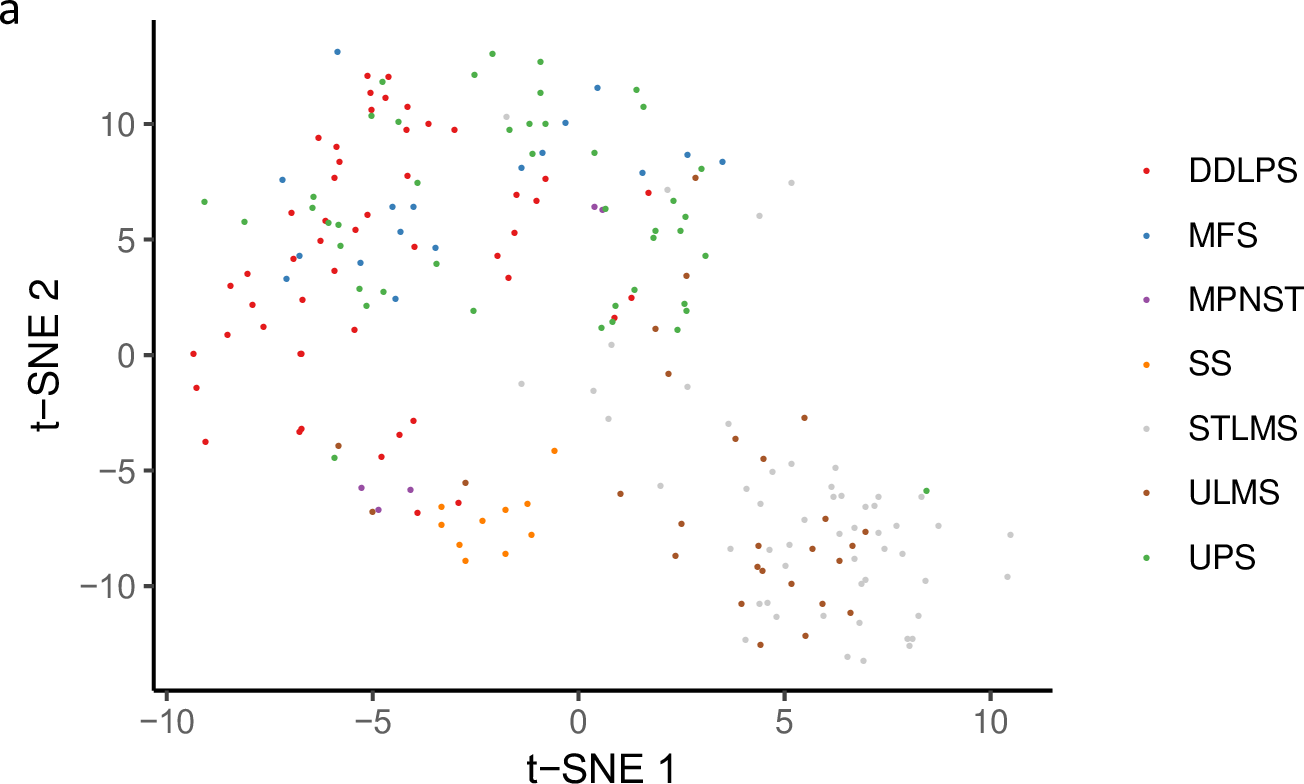

Supplement: S2 Fig — (a) t-SNE analysis of all soft tissue sarcoma samples, colored according to the subtype. (TIF) [file pcbi.1006826.s002.tif]

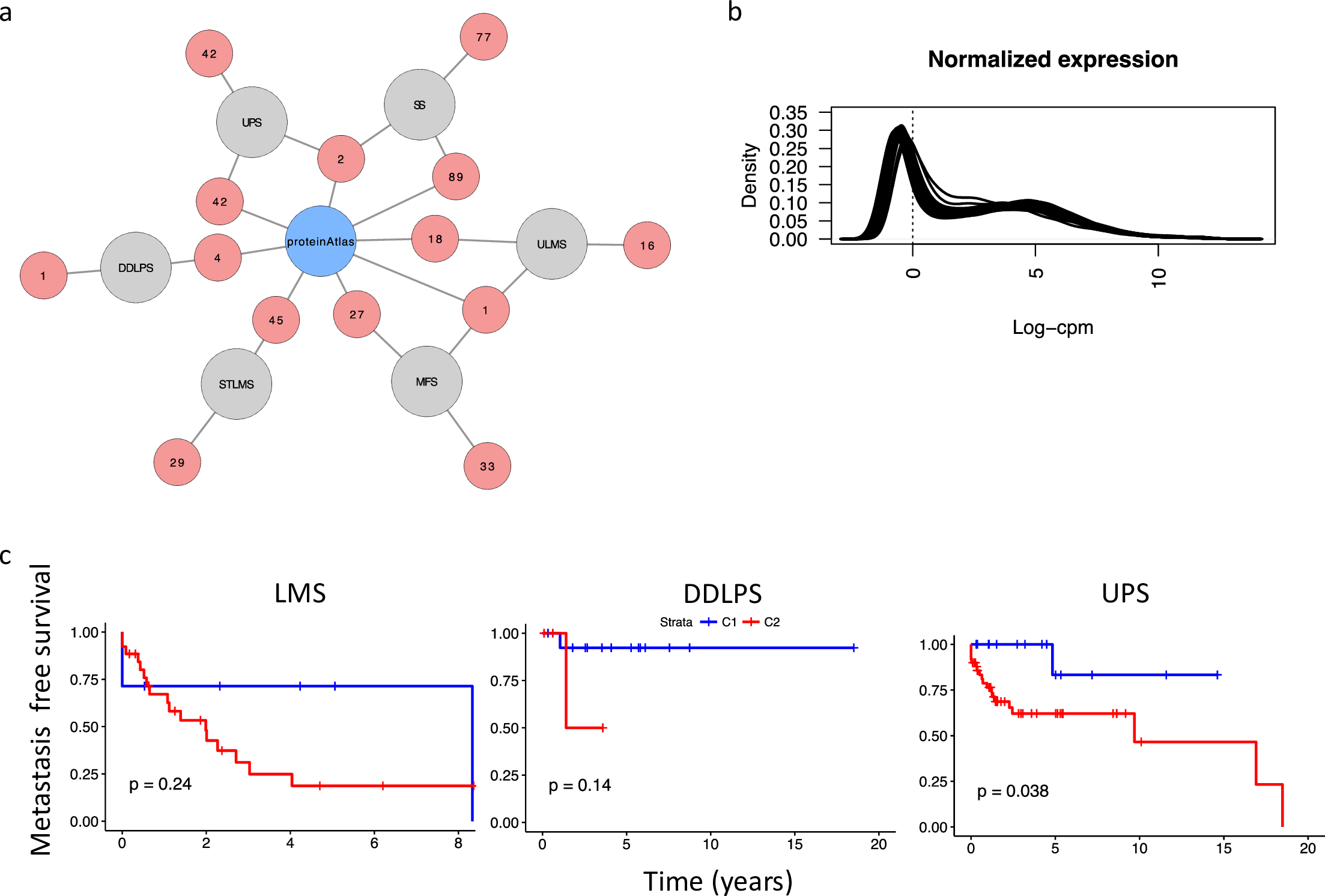

Supplement: S3 Fig — (a) Differences and overlap with the genes that are prognostic, as found in the Pathology Atlas analysis. Many of the identified prognostic genes are also prognostic genes in other cancer types. Number of prognostic genes are shown in the red circles, tumor types in the gray circles and all tumor types analyzed in the protein atlas are shown as a collection in the blue circle. (b) Normalized expression data from the French Sarcoma Group array expression data from sarcomas. (c) Classification according to the CINSARC C1 or C2 classification in the second cohort. (TIF) [file pcbi.1006826.s003.tif]
